# Mg–Zn–Ca Alloy with Ultra-High Ductility and Strength Processed by Screw Rolling

**DOI:** 10.3390/ma18112586

**Published:** 2025-06-01

**Authors:** Haoran Zheng, Weitao Sun, Lijun Deng, Li Zhao, Kwang Seon Shin, Jian Zhang

**Affiliations:** 1 Aerospace and Mechanical College, Shandong University of Aeronautics, Binzhou 256600, China; zhenghaoran916@163.com (H.Z.); sun_wei_tao@163.com (W.S.); denglijun_piston@163.com (L.D.); zhaoliafr@163.com (L.Z.); 2 Shandong Provincial Key Laboratory of Advanced Technology and Equipment for Laser Additive Manufacturing, Binzhou 256600, China; 3 Shandong Key Laboratory of Advanced Engine Piston Assembly, Binzhou 256600, China; 4 Magnestum Technology lnnovation Center, School of Materials Science and Engineering, Seoul National University, Seoul 08826, Republic of Korea

**Keywords:** Mg–Zn–Ca alloy, screw rolling, microstructure, precipitation, mechanical properties

## Abstract

Mg alloys are highly attractive for biodegradable surgical clips because of their low density and good biocompatibility; however, their limited strength and ductility restrict their widespread application. To overcome this limitation, this study employed screw rolling (SR) to produce a Mg–3Zn–0.2Ca alloy with a fine microstructure and an average grain size of 1.6 µm. Experimental results showed that the SR process improved the comprehensive tensile properties of the alloy, increasing the yield strength, ultimate tensile strength, and elongation from 192.6, 234.4 MPa, and 21.7% for the pre-extruded alloy to 252.3, 289 MPa, and 39.5%, respectively. Quantitative analysis of the strengthening behaviour identified grain refinement as the primary strengthening mechanism, along with considerable contributions from Orowan and dislocation strengthening. The ultra-high-tensile ductility was primarily attributed to the low internal stress, nano-sized precipitates, texture weakening, and activation of multiple slip systems. These findings provide a strategy for simultaneously increasing the ductility and strength of Mg alloys and lay a foundation for applying them as biodegradable clips.

## 1. Introduction

Biodegradable clips for laparoscopic surgery have attracted widespread attention because they can avoid additional pain and injury caused by removal. Among the different types of surgical clips, the mechanical strength of biodegradable polymer clips is relatively low, and their structure is relatively complex, which limits their application [1,2]. In recent years, Mg and its alloys have garnered growing attention in the realm of biomedical materials because of their excellent biocompatibility and biodegradability [3,4,5,6]. However, pure Mg has limited clinical viability because of its low ductility and strength, which are attributed to its hexagonal close-packed (HCP) structure [7]. Adding alloying elements or imposing severe plastic deformation are both effective methods for improving these mechanical properties [8].

Research has shown that Mg–Zn–Ca alloys are suitable for such biodegradable clips [9]. Zn and Ca are important minerals for the human body, offering superior compatibility with human tissues compared to Al and rare-earth elements [10,11]. Incorporating Zn and Ca can strengthen Mg alloys through both grain refinement and solid-solution mechanisms; additionally, they form uniformly distributed secondary-phase distributions, which further boost the alloy’s mechanical properties [12]. Moreover, when Zn and Ca are incorporated into Mg alloys, together with the internal stress concentration caused by extrusion, they activate the <c + a> slip, thereby enhancing the ductility of the resultant Mg–Zn–Ca alloy [13].

Meanwhile, some researchers have plastically deformed Mg–Zn–Ca alloys to improve their mechanical properties. For example, a Mg–1.0Zn–0.5Ca alloy extruded at 370 °C demonstrated a high elongation of 44%, but its yield strength (YS, 105 MPa) and ultimate tensile strength (UTS, 205 MPa) were low [14]. By contrast, a Mg–0.6Zn–0.5Ca alloy, processed via double equal channel angular pressing (D-ECAP) at 280 °C, exhibited a higher YS of 372 MPa but a lower elongation of 7% [15]. In addition, significant residual stress occurs at the apex of surgical clips during closure, easily leading to cracking [16]. Therefore, optimising the processing method to improve the plasticity and overcome the strength–ductility trade-off of alloys for such clips is important.

Screw rolling (SR) is an emerging deformation processing method with considerable application potential that can refine alloy grains by inducing a large plastic deformation [17,18,19,20,21]. Notably, SR always produces parts with smooth surfaces, which is a considerable advantage of this method. Further, the area reduction achieved by one rolling pass of an SR mill is approximately equal to that obtained by 6–8 rolling passes in a traditional mill, indicating its higher efficiency [22]. The SR deformation process differs from extrusion or rolling but is similar to torsion deformation. As the rolls rotate to deform the billet, it also moves forward in a spiralling motion, which enables the mill to greatly reduce the billet’s cross-sectional area. The heat generated by this large plastic deformation and friction may cause dynamic recrystallisation (DRX). Thus, the SR process enables the production of materials with fine-grained structures and enhanced mechanical properties [23].

However, to the best of our knowledge, the SR technique has only rarely been applied to Mg alloys [24,25,26]. When pure Mg [24] was processed using SR technology, its YS increased from 55 to 146 MPa after 11 SR passes, demonstrating the efficacy of SR for strengthening Mg. In another study, SR processing increased the YS of a pre-extruded Mg–3.71Zn–2.04Al–0.63Ca–0.62Mn alloy [25] from 225.3 to 272.7 MPa because of grain refinement and the dissolution of the secondary phases; however, its elongation decreased from 23.9 to 8.3%. Meanwhile, the fracture elongation of a Mg–5.56%Zn–0.82%Mn alloy [26] increased from 15.2% for the as-received samples to 27.8% after nine SR passes at 300 °C; this increase of 82.9% was primarily attributed to its microstructure and weakened texture. In addition, SR promoted DRX and decreased the elevated dislocation density that was initially induced by work hardening in the as-received sample. Moreover, the YS increased from 115 to 189 MPa through grain refinement and precipitation strengthening. Notably, the final microstructure and texture of a Mg alloy are influenced not just by the alloying elements but also by the processing parameters, such as the number of processing passes and the processing temperature.

Our previous studies showed that the Mg–3Zn–0.2Ca (wt.%) alloy has good biocompatibility, and its YS and elongation were 205 MPa and 17.85%, respectively [27,28]. Therefore, in this study, a Mg–3Zn–0.2Ca alloy was pre-extruded and then subjected to SR to improve its strength and plasticity. An analysis was conducted on the microstructure, texture, secondary-phase, and mechanical performance of the SR-processed Mg–Zn–Ca alloy. The outcomes offer a fresh strategy for boosting the mechanical properties of Mg-based alloys.

## 2. Experimental Details

**Figure 1:**
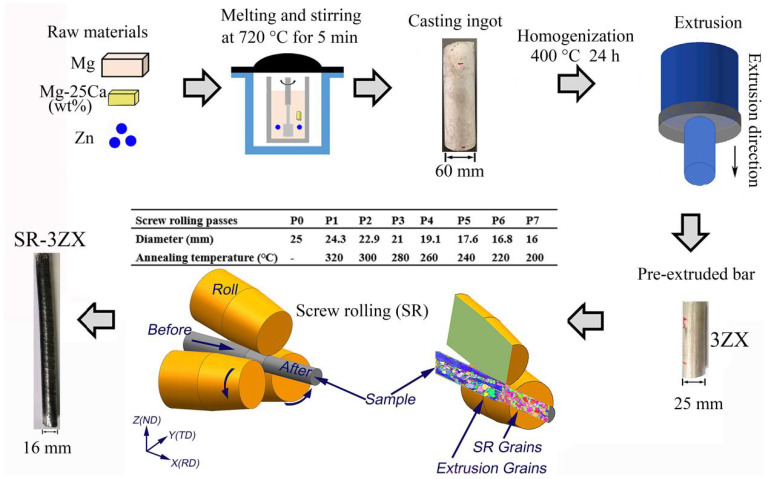
Schematic of the casting, pre-extrusion, and SR processes of the Mg–Zn–Ca alloy.

This study employed a pure Mg (99.99%) ingot, Zn particles (99.99%, Ke Wei company of Tianjin University Co. Ltd., Tianjin, China), and a Mg–25Ca intermediate alloy as the raw materials. The Mg–3Zn–0.2Ca alloy was synthesized using a vacuum induction melting furnace (Shanghai Chenhua Science Technology Co. Ltd., ZG-10, Shanghai, China) in an Ar atmosphere. The raw materials were melted and stirred at 720 °C for 5 min to homogenise the composition before casting in a graphite mould at 690 °C. After cooling naturally in the furnace, rod billets with a 60 mm diameter were produced. Next, they underwent a homogenization process at 400 °C for 24 h. Following this, a hot extrusion was carried out at 320 °C using a YQ32-315 extruder (Shangdong DaYin Industry Machine Co., Ltd., Jinan, China), with an extrusion ratio of 6:1, resulting in a pre-extruded bar measuring ∅25 mm. The SR process [29] was then performed, with a 30-min anneal before each pass in the sequence of 320, 300, 280, 260, 240, 220, and 200 °C [24]. Finally, a ∅16 mm SR bar was obtained. Figure 1 schematically illustrates the process for preparing the alloy. Hereafter, the specimens identified as 3ZX and SR-3ZX correspond to the pre-extruded and SR Mg–3Zn–0.2Ca (wt%) samples, respectively.

To observe the microstructures of the alloy specimens, the test specimens were sectioned perpendicular to the direction of extrusion. The microstructure was examined using optical microscopy (OM; OLYMPUS, U-TV0.5XC-3, Tokyo, Japan), scanning electron microscopy (SEM; Helios NanoLab 460HP, Hillsboro, OR, USA), transmission electron microscopy (TEM; Talos F200X, Thermo Fisher Scientific, Waltham, MA, USA), and electron backscatter diffraction (EBSD; Oxford Instruments, NordlysNano, Oxford, UK). For the EBSD analysis, OIM (https://www.edax.com/products/ebsd/oim-analysis, accessed on 8 May 2025) and ATEX software were employed. The grain size was determined by applying the linear intercept method to the obtained EBSD maps.

**Figure 2:**
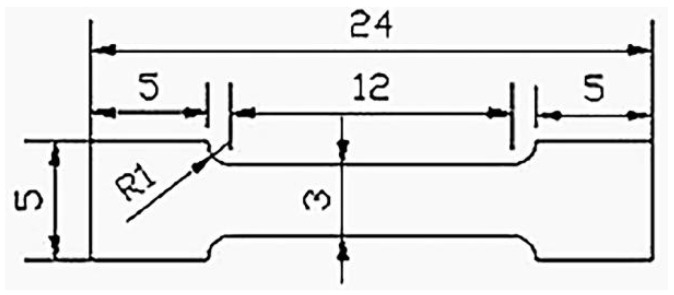
Dimensions of the tensile test samples (mm).

The hardness values of the specimens were measured using a Vickers hardness tester (HMV-2T, Shimadzu, Kyoto, Japan) with a load of 9.8 N for 20 s. Each specimen was randomly tested in at least six locations, and the values were averaged. Specimens for tensile testing were crafted from a location 0.4 mm from the edge of the bar in the extrusion direction, following the dimensions depicted in Figure 2. The mechanical performance was assessed by tensile tests using a DNS universal testing machine with a strain rate of 10^−4^ s^−1^ at ambient temperature. To ensure accuracy, three identical samples were tested. The fracture surface was observed via SEM, and energy-dispersive X-ray spectroscopy (EDS) was used to analyse the chemical composition of the sample and fracture surfaces. A typical TEM dual-beam analysis was carried out to identify the types of dislocation activation.

## 3. Results

### 3.1. Tensile Properties

**Figure 3:**
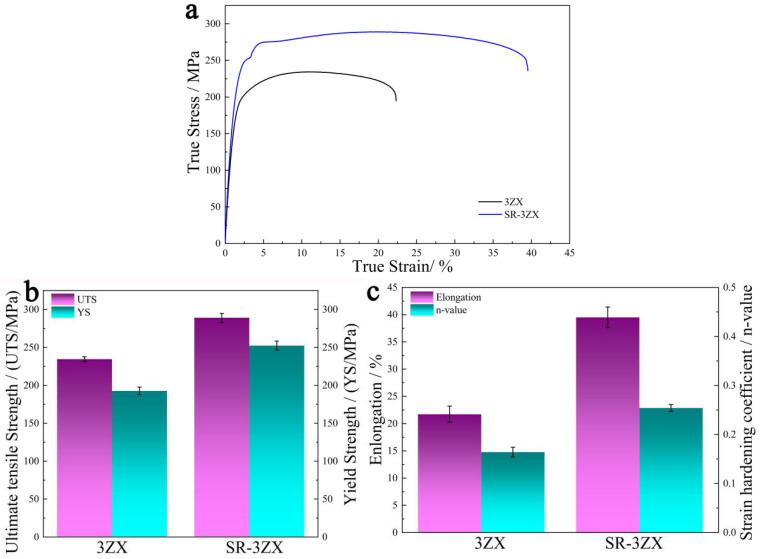
(**a**) Tensile stress–strain curves and the (**b**,**c**) corresponding mechanical properties of 3ZX and SR-3ZX: (**b**) UTS (left axis) and YS (right axis), as well as (**c**) elongation (left axis) and strain hardening coefficient (right axis). The error bars indicate the standard deviation (n = 3).

Figure 3a illustrates the true stress–strain curves for 3ZX and SR-3ZX, and Figure 3b,c plot their corresponding mechanical performance parameters. The 3ZX specimen exhibits a low UTS of ~234.4 MPa, YS of ~192.6 MPa, and elongation of ~21.7%. Remarkably, for SR-3ZX, these values increase simultaneously to ~289 MPa, ~252.3 MPa, and ~39.5%, respectively. Furthermore, the strain hardening coefficients (*n*) of 3ZX and SR-3ZX are 0.16 and 0.25, respectively. An increased value of n indicates that the material is capable of withstanding greater strain hardening [30,31]; thus, SR-3ZX also exhibits superior plasticity. In addition, the hardness of 3ZX increased from ~70 to ~82 HV after SR. Overall, SR-3ZX demonstrates favourable mechanical performance, successfully balancing robust strength with remarkable ductility.

**Figure 4:**
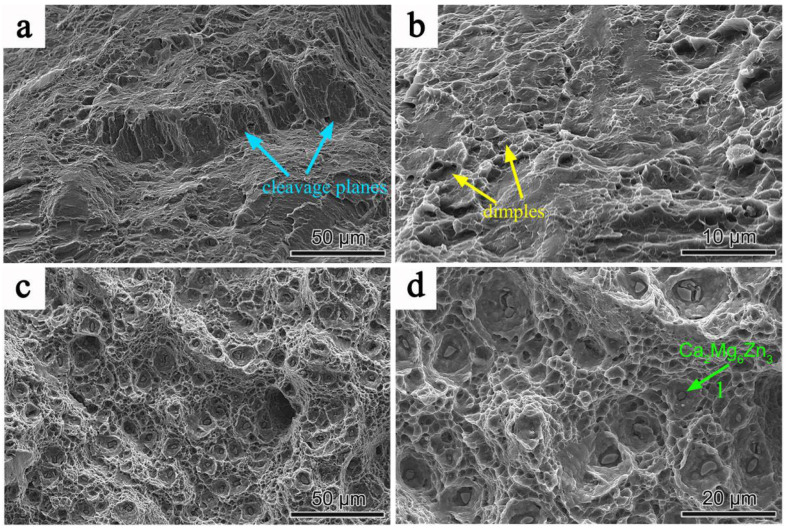
SEM images of fracture surfaces of (**a**,**b**) 3ZX and (**c**,**d**) SR-3ZX.

Figure 4 shows the fracture surfaces of 3ZX and SR-3ZX following tensile tests conducted at room temperature. As shown in Figure 4a,b, the 3ZX fracture surface exhibits quasi-cleavage fracture characteristics, containing cleavage planes (blue arrows) and a few dimples (yellow arrows). However, as shown in Figure 4c,d, the fracture morphology of SR-3ZX exhibits microvoid coalescence, as evidenced by the presence of many dimples and the absence of typical cleavage planes. Compared with those of SR-3ZX, the 3ZX dimples are smaller and shallower. These phenomena confirm that the SR process significantly increases the ductility of the alloy, as demonstrated by these typical features of a ductile fracture surface. In addition, the green arrow in Figure 4d shows that the precipitates are uniformly distributed at the bottoms of the dimples in the SR-3ZX fracture surface. Further, EDS analysis reveals that the chemical composition of point 1 indicated by the green arrow is 69.87 at.% Mg, 17.62 at.% Zn, and 12.51 at.% Ca, implying that these particles are Ca_2_Mg_6_Zn_3_ precipitates. Such nanoscale Ca_2_Mg_6_Zn_3_ precipitates not only hinder dislocation movement but also promote dislocation multiplication [32], which facilitates dimple formation and improves the material toughness. Overall, the fracture surface clearly indicates the remarkable ductility of the SR-3ZX alloy.

### 3.2. Microstructures

**Figure 5:**
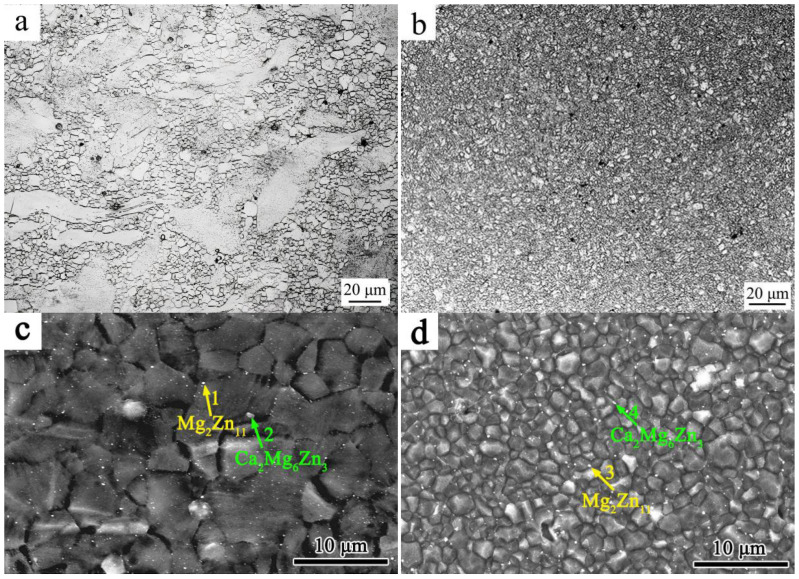
(**a**,**b**) Optical and (**c**,**d**) SEM images of the microstructures of (**a**,**c**) 3ZX and (**b**,**d**) SR-3ZX.

Figure 5 shows optical images and transverse cross-sectional SEM images of the microstructures of 3ZX and SR-3ZX. Figure 5a,b reveal that, unlike SR-3ZX, which comprises ultra-fine equiaxed DRX grains, the 3ZX structure contains both the original coarse grains and fine equiaxed grains. This indicates that the shear strain induced by SR provides sufficient energy for DRX. As shown in Figure 5c,d, many precipitates are uniformly distributed in both alloys. By combining the phase diagram with the EDS results from points 1–4 in Figure 5c,d, which are summarised in Table 1, these precipitates are determined to be Ca_2_Mg_6_Zn_3_ and Mg_2_Zn_11_.

**Figure 6:**
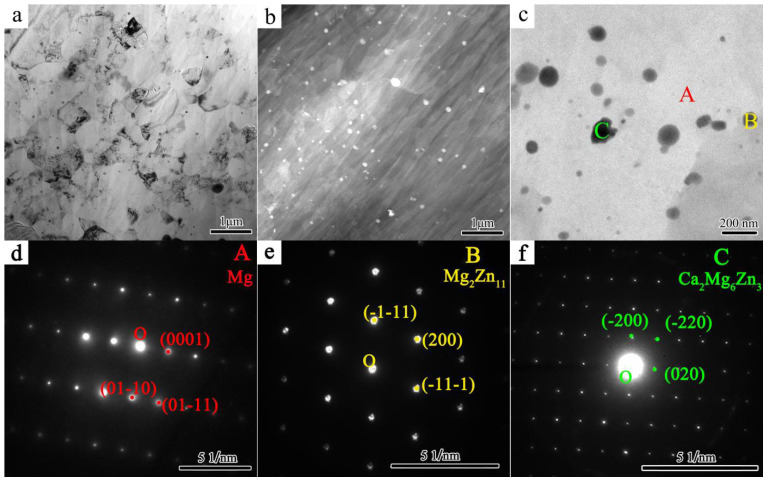
TEM images of SR-3ZX. (**a**) Bright-field (BF), (**b**–**d**) HAADF, and (**c**) precipitate phase images; (**d**–**f**) diffraction spots of points A–C in (**c**).

On average, the SR-3ZX grains are significantly smaller and more uniform in size than the 3ZX grains. To further observe the SR-3ZX microstructure, TEM images were obtained (Figure 6). The bright-field (BF) TEM image of SR-3ZX in Figure 6a displays lattice distortion within the grains, which appears black in this image. The high-angle annular dark-field (HAADF) image of SR-3ZX in Figure 6b reveals a large amount of nanoscale precipitates with an average diameter of ~34 nm. The average interparticle spacing was determined to be ~338 nm by the linear intercept method. In SR-3ZX, these precipitates are evenly spread throughout both the grain boundaries and the interiors of the grains. Selected area electron diffraction (SAED) patterns were recorded from points A–C in Figure 6c, which identified the phases as α-Mg, Mg_2_Zn_11_, and Ca_2_Mg_6_Zn_3_, respectively (Figure 6d–f). These findings are consistent with the SEM results in Figure 5.

**Figure 7:**
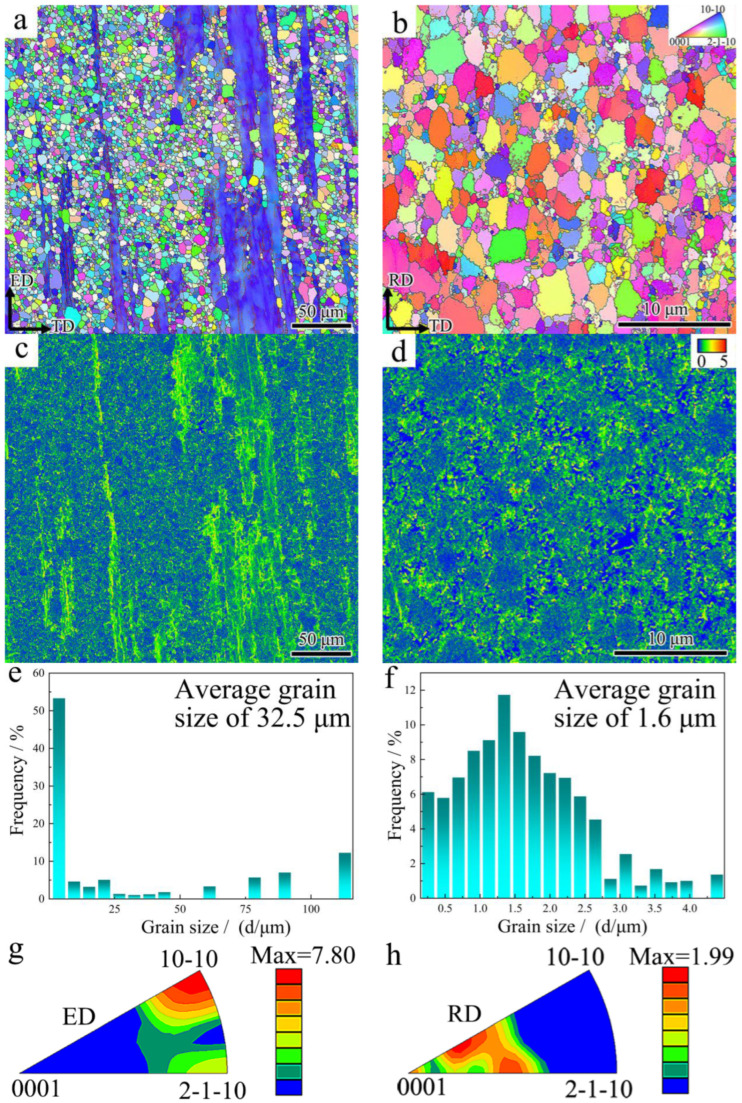
EBSD results for 3ZX (**a**,**c**,**e**,**g**) and SR-3ZX (**b**,**d**,**f**,**h**): (**a**,**b**) IPF maps, (**c**,**d**) KAM maps, (**e**,**f**) grain size distributions, and (**g**,**h**) IPFs.

Figure 7 shows the EBSD results for 3ZX and SR-3ZX. The inverse pole figure (IPF) maps of 3ZX and SR-3ZX in Figure 7a,b, respectively, reveal that SR reconfigures the grain boundaries. The 3ZX specimen exhibits a distinct bimodal microstructure comprising equiaxed recrystallised grains with diameters of several micrometres and non-recrystallised regions with coarse elongated grains. By contrast, the SR-3ZX sample is fully recrystallised. In addition, the grains are more randomly orientated in the SR sample, as evidenced by the more-randomly coloured grains in its IPF (Figure 7a). Furthermore, as shown in Figure 7e,f, the average grain sizes of 3ZX and SR-3ZX are 32.5 and 1.6 μm, respectively, corresponding to a reduction of 95.1% after SR. The corresponding kernel average misorientation (KAM) map in Figure 7c indicates that, in the non-recrystallised region of 3ZX, more severe lattice distortion is retained than in the recrystallised region; this contributes to a high geometrically necessary dislocation (GND) density of 1.21 × 10^14^ m^−2^, which was calculated using ATEX software based on the EBSD data. For SR-3ZX (Figure 7d), however, the residual stress distribution is more uniform, and the high-stress regions are smaller. Further, the GND density decreases to 5.35 × 10^13^ m^−2^. This indicates that the internal stress and high density of dislocations in the material are released via recrystallisation during the SR process, making the internal stress in SR-3ZX lower than that of 3ZX. The IPF for 3ZX (Figure 7g) reveals a bimodal texture (similar to a rare-earth texture), indicating the formation of a non-basal texture during the hot extrusion procedure. Following SR, however, the grain boundary misorientation angle of SR-3ZX (Figure 7h) is deflected, forming a similar <10−12>//RD texture [33,34]. Compared with that of 3ZX, the maximum texture intensity of SR-3ZX decreases from 7.80 to 1.99, implying superior plasticity.

**Figure 8:**
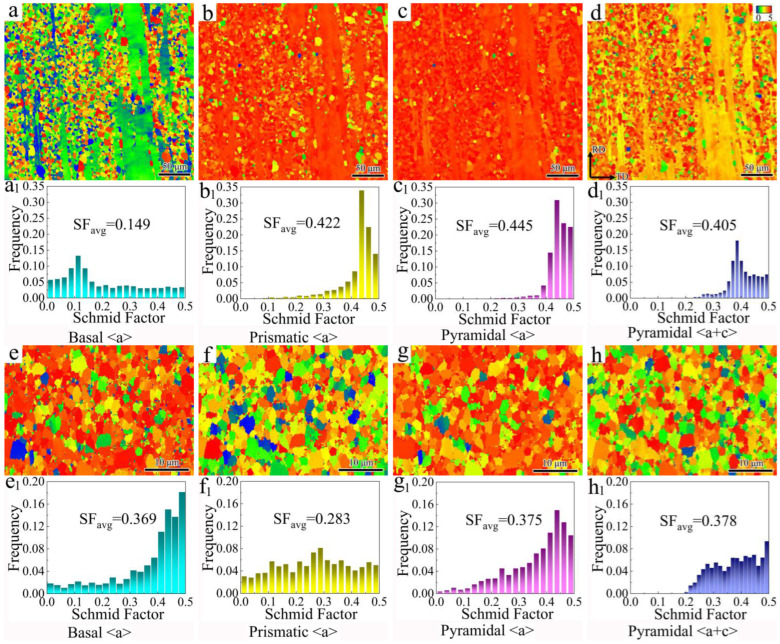
SF maps for various slip systems in 3ZX (**a**–**d**) and SR-3ZX (**e**–**h**) (basal: {0 0 0 1}<1 1 −2 0>, prismatic: {1 0 −1 0}<1 1 −2 0>, pyramidal: {1 0 −1 1}<1 1 −2 0>, pyramidal: {1 1 −2 2}<1 1 −2 3>), and corresponding histograms illustrated in (a1–h1), respectively.

Figure 8 shows the Schmid factors (SFs) of different slip systems in 3ZX and SR-3ZX. In 3ZX, the SF of the basal <*a*> slip is lower than those of the prismatic <*a*>, pyramidal <*a*>, and pyramidal <*a* + *c*> slips, indicating that the activation of the non-basal slip systems is more likely. By contrast, the SFs of the different slip systems in SR-3ZX do not differ significantly, indicating that more slip systems may have been activated. In addition, the average SF of the basal slip increases after SR, which improves the material ductility.

## 4. Discussion

**Figure 9:**
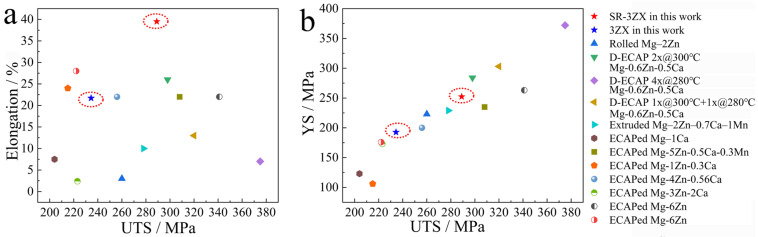
Comparison of the mechanical properties of other biomedical Mg–Zn alloys with those prepared in this study: (**a**) Values of UTS and elongation, and (**b**) values of UTS and YS.

Figure 9 presents a comparison of the mechanical properties of the materials assessed in this study with those of other biomedical Mg–Zn alloys [35,36,37,38,39,40,41]. Notably, the SR-3ZX alloy produced in this work is considerably more ductile than all the other Mg–Zn alloys (Figure 9a), but its strength is comparable to many of the high-strength Mg–Zn alloys (Figure 9b). Thus, the SR processing of these alloys overcomes the strength–ductility trade-off found in previously documented Mg–Zn alloys. The elevated ductility indicates the enhanced formability of the Mg alloy at ambient temperature, highlighting the significant potential of SR-3ZX as a Mg-based biodegradable clip.

During SR plastic deformation, the bar experiences uniform stress in three directions and moves forward while spiralling due to the frictional force between the roll and the bar. Throughout several passes, the bar undergoes torsion deformation, leading to a decrease in diameter and an increase in length. As deformation occurs, the external shear and compressive stresses gradually infiltrate the metal bar, causing metal flow and plastic deformation, which subsequently refine the grains within the microstructure. In particular, the strain energy generated by SR encourages grain nucleation for DRX, leading to notable grain refinement of SR-3ZX under the influence of dual-stress conditions.

The influence of grain refinement on the YS can be assessed through the Hall–Petch relationship. In addition, residual dislocations can enhance the strength. The presence of GNDs impedes dislocation activation during plastic deformation, thereby enhancing the YS of SR-3ZX. Furthermore, the Orowan mechanism, which refers to dislocations bowing around obstacles such as precipitates, can also strengthen the material. In SR-3ZX, the high number fraction of nanoscale secondary phases and the short distance between these precipitates substantially increased the degree of Orowan strengthening.

To further quantify the contributions of the grain boundary strengthening (σ_GB_), Orowan strengthening (σ_Orowan_), and dislocation strengthening (σ_Dislocation_) mechanisms to the YS of SR-3ZX, the theoretical YS value of each possible strengthening mechanism was calculated using the following equations [42,43,44,45,46,47,48]:

σ_GB_ = σ_0_ + *kd*^−1/2^, (1)

(2) $$\sigma_{Orowan}=\frac{0.4MGb}{\pi \lambda }ln\frac{d1}{b}/\sqrt{1-\nu_{Mg\;}},$$

(3) $$\sigma_{Dislocation}=M\alpha Gb\sqrt{\rho_{Dislocation}},$$

where *σ*_0_ is 44 MPa for the Mg–Zn–Ca alloy [13]; *k* represents the slope of the Hall–Petch relation, with a value of 153 MPa μm^1/2^; *d* refers to the average grain size of SR-3ZX; the value of the Taylor factor, symbolized as *M*, is 3.5; the shear modulus, indicated by *G*, is 17 GPa for the Mg–Zn–Ca alloy; the Burgers vector, symbolized as *b*, has a magnitude of 0.32 nm; *λ* refers to the average interparticle spacing; and the effective diameter of the precipitates is indicated by *d*_1_. For magnesium, Poisson’s ratio, denoted as *ν*_Mg_, is 0.35. The numerical constant α, which is 0.2, assesses the efficiency of dislocation strengthening. The total dislocation density, represented by *ρ*_Dislocation_, is approximately 1.5 times greater than the GND density.

**Figure 10:**
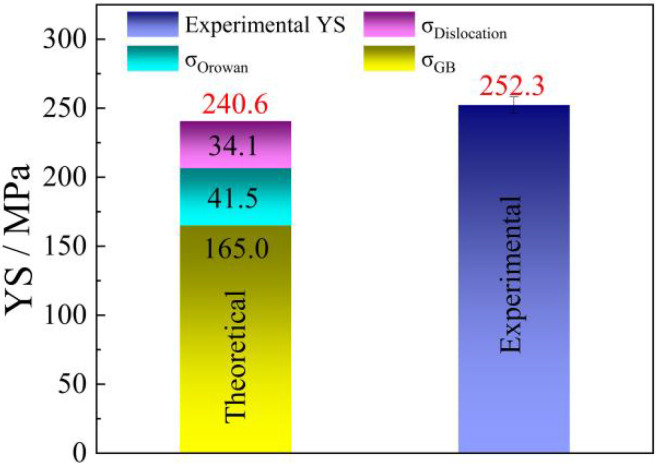
Quantified contributions of different strengthening mechanisms and a comparison of the theoretical YS with the experimentally determined YS of SR-3ZX, where the theoretical YS (σ_Theory_) = σ_GB_ + σ_Orowan_ + σ_Dislocation_.

The contributions of σ_GB_, σ_Orowan_, and σ_Dislocation_ to the YS of SR-3ZX were calculated to be 165.0, 41.5, and 34.1 MPa, respectively. The contributions of the various strengthening mechanisms and a comparison with the experimental results are shown in Figure 10. The predicted YS was 240.6 MPa, which is slightly lower than the experimental YS value of ~252.3 MPa. This discrepancy can possibly be attributed to texture strengthening, as the texture was not high and therefore was not considered in the analysis. Nonetheless, the three aforementioned strengthening mechanisms could be concluded to synergistically strengthen SR-3ZX [49,50,51]. Grain refinement was the main mechanism for strengthening in SR-3ZX; however, the contributions of the Orowan and dislocation strengthening mechanisms were also not trivial.

**Figure 11:**
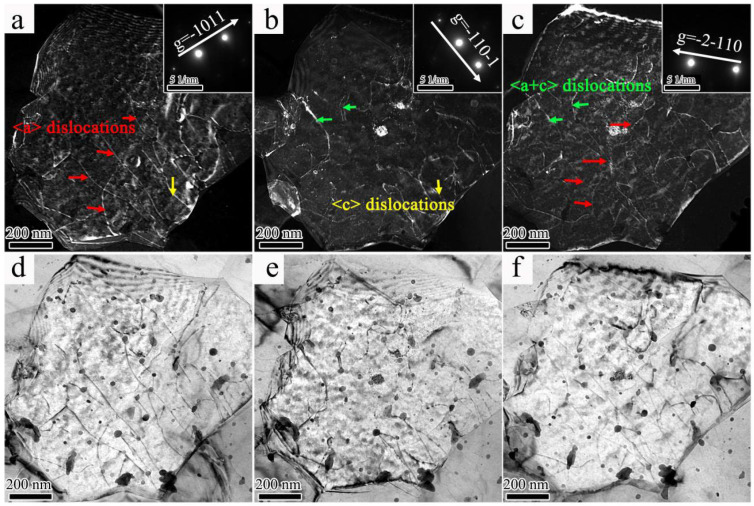
DF (**a**–**c**) and corresponding BF TEM images (**d**–**f**) of SR-3ZX under *g* = [−1 0 1 1] (**a**,**d**), *g* = [−1 1 0 −1] (**b**,**e**), and *g* = [−2 −1 1 0] (**c**,**f**). The red, orange, and green arrows indicate <*a*>, <*c*>, and <*a* + *c*> dislocations.

Additionally, SR-3ZX showed exceptional ductility, achieving a fracture elongation reaching 39.5%. To explore the properties underlying this exceptional ductility, the TEM results used to identify the activated dislocations of SR-3ZX under the double-beam condition are shown in Figure 11. Figure 11a,b present BF and dark-field (DF) images under the two-beam condition of *g* = [–1011], respectively. Numerous <*a*> dislocations (marked in red) and a small number of <*c*> dislocations (yellow) are clearly identified. The <*c*> and <*c* + *a*> dislocations (green) are observed under *g* = [–110–1] (Figure 11b). As shown in Figure 11c,f, under the *g* = [–2–110] vector, <*a*> dislocations are mainly observed, accompanied by a small number of <*c* + *a*> dislocations. These results indicate that <*a*>, <*c*>, and <*c* + *a*> dislocations can all be activated in SR-3ZX. For HCP metals, the scarcity of slip systems often results in insufficient ductility. Therefore, the high plasticity of SR-3ZX is due to the widespread activation of slip systems. The BF TEM images (Figure 11d–f) reveal that a large number of ultra-fine nanoprecipitates pin the dislocations, and the activation of various slip systems increases the number of dislocation intersections caused by different dislocation movements. These enhance the resistance to dislocation movement through SR-3ZX, thereby improving its strength.

Under SR, the bimodal texture of 3ZX changed to a more uniform <10−12>//RD texture in SR-3ZX because of the large plastic deformation and DRX induced by the SR process. Additionally, the texture weakened. Therefore, the stress concentration could be more easily released, as evidenced by the higher fracture elongation of SR-3ZX. Texture weakening, therefore, contributed to improving the plasticity of SR-3ZX. Moreover, uniformly distributed nanoprecipitates in SR-3ZX (Figure 6) promoted dislocation accumulation, yielding a high ductility [52,53,54]. In addition, the lower dislocation density in SR-3ZX (Figure 7d) provided additional space for dislocation sliding and accumulation during deformation, which may have reduced the work-hardening capability and improved ductility [55,56]. Finally, the initiation of basal and non-basal slip systems provided more dislocations (Figure 11) to promote plastic deformation; thus, the SR-3ZX strain hardening (Figure 3) and ductility were improved by the SR-induced deformation.

This study demonstrated that SR-3ZX has excellent ductility, showing great promise for fabricating biodegradable surgical clips. However, we only studied its mechanical properties; hence, future studies will focus on carefully investigating its corrosion resistance and biocompatibility as well as optimising its structure.

## 5. Conclusions

The microstructure, grain refinement, precipitates, texture, and tensile properties of a biomedical Mg–Zn–Ca alloy processed by SR were examined in this study. Under SR, the average grain size decreased significantly from 32.5 μm for 3ZX to 1.6 μm for SR-3ZX. Moreover, SR-3ZX exhibited an ultra-high elongation of ~39.5%, an excellent YS of ~252.3 MPa, and a UTS of ~289 MPa, which were 82.0%, 31%, and 23.3% higher, respectively, than those of 3ZX prior to SR. The high YS of SR-3ZX primarily stemmed from grain boundary strengthening (~165 MPa), with the considerable involvement of Orowan strengthening (~41.5 MPa) induced by precipitates, as well as dislocation strengthening (~34.1 MPa). The weak internal stress, nanoscale precipitates, texture weakening, and activation of multiple slip systems were the main factors generating the ultra-high tensile ductility of SR-3ZX up to ~39.5%. These metrics make SR-3ZX a promising Mg alloy for application in biodegradable clips.

## Figures and Tables

**Table 1:** Chemical compositions (at.%) of points 1–4 in Figure 5 as determined via EDS.

Location	Mg	Ca	Zn
1	58.66	0.97	40.37
2	64.05	15.03	20.92
3	59.83	1.01	39.16
4	61.82	14.33	23.85

## Data Availability

The raw/processed data required to reproduce these findings cannot be shared.
